# mir-660-p53-mir-486 Network: A New Key Regulatory Pathway in Lung Tumorigenesis

**DOI:** 10.3390/ijms18010222

**Published:** 2017-01-23

**Authors:** Cristina Borzi, Linda Calzolari, Giovanni Centonze, Massimo Milione, Gabriella Sozzi, Orazio Fortunato

**Affiliations:** 1 Department of Experimental Oncology and Molecular Medicine, Unit of Tumor Genomics, Fondazione IRCCS Istituto Nazionale dei Tumori, 20133 Milan, Italy; cristina.borzi@istitutotumori.mi.it (C.B.); linda.calzolari@istitutotumori.mi.it (L.C.); giovanni.centonze@istitutotumori.mi.it (G.C.); gabriella.sozzi@istitutotumori.mi.it (G.S.); 2 Department of Pathology and Laboratory Medicine, Fondazione IRCCS Istituto Nazionale dei Tumori, 20133 Milan, Italy; massimo.milione@istitutotumori.mi.it

**Keywords:** miRNAs, lung cancer, p53

## Abstract

Lung cancer is the most frequent cause of cancer-related death worldwide, with limited therapeutic options and rapid development of drug resistance. MicroRNAs, a class of small non-coding RNAs that control different physiological processes, have been associated with cancer development, as either oncomiRNAs or tumor-suppressor miRNAs. In the present study we investigated the interaction between mir-486-5p and mir-660-5p, two independent tumor-suppressor miRNAs, to assess their possible role and synergistic effect in lung cancer treatment. Our data show that mir-660-5p over-expression in A549 lung cancer cells induced a remarkable increase in mir-486-5p expression level and activity, detected as a reduction of its target gene, p85. mir-486-5p expression was confirmed by microRNA in situ hybridization. mir-660-5p modulated mir-486-5p through the silencing of Mouse Double Minute 2 (MDM2), one of its direct target, and then through p53 stimulation. This regulatory pathway was effective in A549, but not in H1299; therefore, only in the context of a functional p53 protein. Our findings support the conclusion that mir-486-5p is positively regulated by mir-660-5p in lung cancer cell lines, through the mir-660-MDM2-p53 pathway, making mir-660-5p even more interesting for its potential successful use in lung cancer therapy.

## 1. Introduction

Lung cancer is the leading cause of cancer-related deaths in the world, accounting for over 20% of all cancer deaths in Europe [1] and with a five-year relative survival estimate around 18% [2]. Although several efforts have been made in reducing lung cancer incidence, through advances in prevention and early detection, lung tumors are still expected to account for approximately 13% of all new cancer diagnoses in 2016 [2]. Personalized therapies tested in clinical trials have shown positive results for non-small cell lung cancer (NSCLC) with advanced disease, however nowadays a large number of patients still remain without any potential known molecular target for therapy [3]. Therefore, there is a great need to further increase the understanding of molecular disease mechanisms, to develop new targeted interventions and more effective drugs [4].

MicroRNAs (miRNAs) are small non-coding RNAs that play a critical role in the regulation of gene expression in cells. They typically inhibit the stability of messenger RNA (mRNA) or its translation by binding to the 3′-UTRs of specific target mRNAs and thereby regulate the expression of genes at the post-transcriptional level [5,6]. A single miRNA can have multiple targets and, therefore, miRNAs could regulate a large number of protein-coding genes included in various signaling pathways [7].

MiRNAs are involved in the majority of canonical cellular processes, such as differentiation, proliferation, survival and metabolism. Deregulation of these biological processes is often implicated in tumorigenesis [8,9]. Thus, alteration of miRNA expression could lead to cancer development, as well as being documented in various human malignancies [10,11]. In this regard, depending on their target genes, miRNAs may function as either oncogenes or tumor suppressors [12].

The *TP53* tumor suppressor is the most frequently mutated gene in human tumors [13]. Of note, the tumor suppressive functions of p53 could contribute to tumor development if not properly regulated [14,15]. For instance, p53 is frequently inactivated by amplification and over-expression of its negative regulator Mouse Double Minute 2 MDM2 in many types of tumors, such as sarcoma, breast cancer and pediatric acute lymphoblastic leukemia [16,17].

Several studies indicate that p53 tumor suppressor activity is frequently inactivated by mutations in NSCLC patients [18,19] or by interaction with MDM2, which eliminates wild-type p53 [20].

Recent studies have revealed interactions between p53 and miRNAs. As a key transcription factor, p53 could directly regulate the expression of selected miRNAs, such as the mir-34 family (mir-34a, mir-34b, and mir-34c), which is involved in cell-cycle arrest or cell death [14,21].

In the context of lung cancer, mir-34a is a target of p53 in NSCLC cells [22]. Concerning this, it has been recently shown that p53 can regulate the PDL1 (Programmed Death 1 Ligand 1) expression level via mir-34a and, together, define a novel mechanism of tumor immune evasion for NSCLC [23].

In addition to mir-34a, p53 protein promotes the expression of other miRNAs in lung cancer cells, including mir-184, mir-148, and mir-181. The last two are onco-suppressor miRNAs that regulate the expression of Cell Division Cycle 73 (CDC73) and Cyclin-dependent kinase 1 (CDK1), respectively, which are proteins involved in the G1 phase of the cell cycle [24].

On the other hand, the expression and activity of p53, itself, is under the control of miRNAs. To date, at least twenty miRNAs have been identified as negative regulators of p53 [25,26]. In NSCLC, it has been shown that p53 is a direct target of mir-150 [24], mir-453, and mir-98, which are involved in cisplatin-induced lung cancer cells death [27].

In addition to the negative regulation of the p53 protein by miRNAs, p53 can be indirectly regulated by those miRNAs that target its regulators, such as MDM2, SIRT1, and cyclin G1 [21,28]. Concerning this, we recently identified mir-660-5p as an additional miRNA that activates p53 through direct binding to the 3′-UTR of MDM2 mRNA [29]. mir-660-5p expression was significantly down-regulated in lung tumors compared with normal lung tissues and mir-660-5p replacement in lung cancer cell lines reduced MDM2 protein levels and increased p53 protein levels, leading to the rescue of p53 functions in inhibiting tumor growth.

In a previous study, our group reported that mir-486-5p was strongly down-regulated in primary lung tumors compared to paired normal tissues [30]. Of note, recently mir-486-5p has been reported as a tumor suppressor in lung cancer directly targeting components of insulin growth factor (IGF) signaling, such as p85α, and its expression has been proved to be regulated by p53 [31]. Based on our data that demonstrate an important role of mir-660-5p in lung tumor suppression through the regulation of the p53/MDM2 negative feedback loop, we here investigate the relationship among mir-660-5p, p53 and mir-486-5p and their role in lung tumorigenesis.

## 2. Results

### 2.1. mir-486-5p Expression Level and Activity Are Positively Regulated by mir-660-5p in Lung Cancer Cells

**Figure 1:**
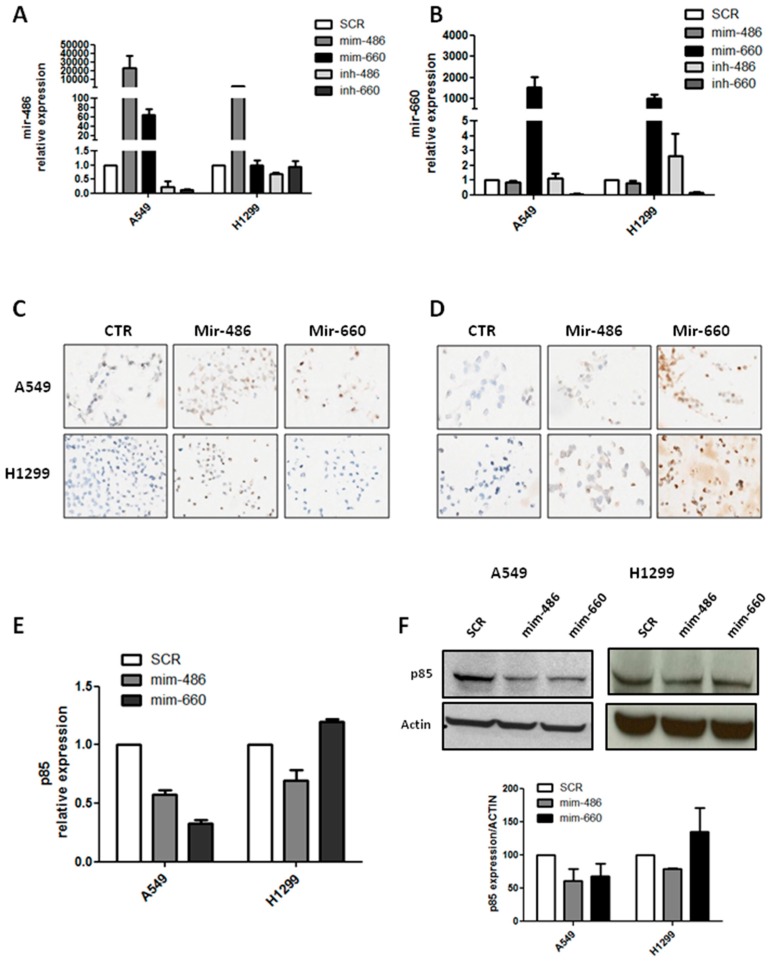
Mir-660-5p over-expression in human lung cancer cell lines regulates mir-486-5p expression and functionality. (**A**) Relative expression of mir-486-5p after transfection with mir-486-5p or mir-660-5p mimic/inhibitor or control in A549 and H1299; (**B**) mir-660-5p expression after transfection with mir-486-5p or mir-660-5p mimic/inhibitor or control in A549 and H1299; (**C**,**D**) representative images of mir-486-5p (**C**) and mir-660-5p (**D**) detection in cell-blocks of lung cancer cells. Original magnification: 20×; (**E**) graphs showing p85 transcript levels after miRNA restoration; (**F**) representative images of p85 protein expression in lung cancer cell lines and Western blot band quantification. All data are expressed as mean ± standard error of the mean (SEM) (*n* = 2 for each cell).

To investigate the relationship between mir-486-5p and mir-660-5p in lung cancer cells, we transiently transfected two lung cancer cell lines with different genotypes (A549 and H1299), p53 wild-type and null, respectively, using commercially available miRNA mimics or inhibitors. Following mir-660-5p over-expression, a great increase in mir-486-5p expression level in A549, about 60-fold compared to control, was observed. In H1299 cell lines mir-486-5p levels were unaffected after mir-660-5p over-expression (Figure 1A). No significant change was observed in mir-660-5p expression upon mir-486-5p modulation, neither in A549 nor in H1299 cell lines (Figure 1B). Interestingly, mir-660-5p knockdown showed a reduction of mir-486-5p expression in A549, whereas this effect was absent in H1299 cells (Figure 1A). These results suggest that, in lung cancer cell lines, mir-660-5p could regulate mir-486-5p expression level through a p53 dependent mechanism.

Moreover, to confirm mir-660-5p and mir-486-5p mutual regulation, in situ hybridization (ISH) on cell-blocks of A549 and H1299 characterized by the over-expression of the two miRNAs was performed. As expected mir-486-5p staining was clearly visible inside both A549 and H1299 cells transfected with the mir-486-5p mimic, but also A549 cell line over-expressing mir-660-5p was positive for miR-486-5p ISH. On the contrary, H1299-mir-660-5p did not show positive staining for mir-486-5p (Figure 1C). Furthermore, ISH for mir-660-5p was positive only on A549 and H1299 over-expressing mir-660-5p areas, whereas A549 and H1299 over-expressing mir-486-5p were both negative (Figure 1D). Thus, ISH results confirmed quantitative real-time PCR data.

Next, p85 expression level, a direct target of mir-486-5p, was measured in order to verify the activation of mir-486-5p signaling in different conditions. When mir-486-5p was over-expressed in cells, the p85 mRNA levels decreased in either A549 or H1299 (about 40% and 30% of reduction, respectively, compared to control) (Figure 1E) and p85 protein levels decreased accordingly (40% reduction for A549 and 22% for H1299, compared to control) (Figure 1F). In addition, the p85 transcript and protein were both negatively regulated by mir-660-5p over-expression in A549 cells (65% reduction of mRNA and 30% of protein levels), as shown in Figure 1E,F. No reduction in p85 expression level was measured in H1299 after mir-660-5p replacement (Figure 1E,F).

Therefore we demonstrated that mir-486-5p is positively modulated and activated by mir-660-5p restoration in lung cancer cell lines, but only in a context of a functional p53 pathway.

### 2.2. mir-486-5p Modulation Is Important for mir-660-5p Antitumoral Effects

To demonstrate that mir-486-5p up-regulation is fundamental for mir-660-5p anti-tumoral effects we inhibited mir-486-5p after mir-660 over-expression, then migration and proliferation in both lung cancer cell lines were analyzed.

**Figure 2:**
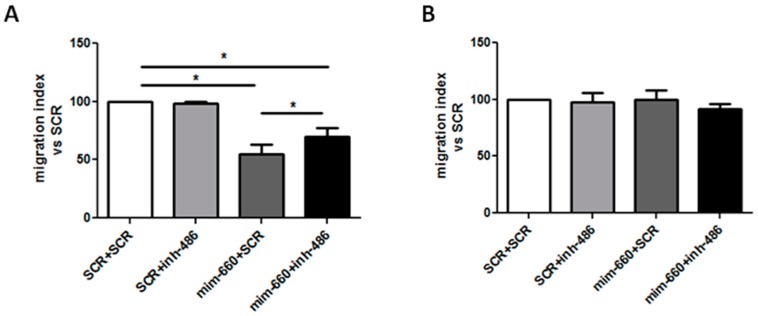

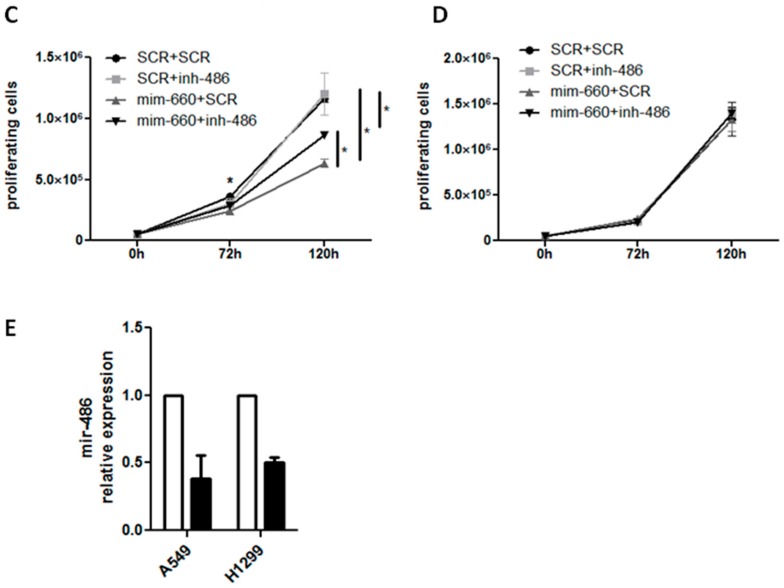
mir-486-5p is critical for mir-660-5p anti-tumoral effects in lung cancer cells. (**A**,**B**) Graphs showing A549 (**A**) and H1299 (**B**) migration in a Transwell assay after mir-660-5p over-expression and/or mir-486-5p inhibition (*n* = 3). Migration is expressed as the number of migrated cells for each transfection vs. the number of migrated control cells; (**C**,**D**) graphs show the number of proliferating A549 (**C**) and H1299 (**D**) cells transfected with mir-660-5p mimic and/or mir-486-5p inhibitor or control and viable cells were counted with trypan blue at 72 and 120 h (*n* = 3); (**E**) mir-486-5p expression levels after locked nucleic acid (LNA) inhibition (black bars), compared to untreated controls (white bars). All data are expressed as mean ± SEM. * *p* < 0.05 vs. controls.

Interestingly, mir-660-5p inhibited migratory ability of A549 cells, compared to control (migration reduction: 45% compared to control, *p* < 0.05) but following mir-486-5p inhibition, the anti-migratory effect of A549 miR-660-5p over-expressing cells were partially diminished (migration reduction: 30% compared to control, *p* < 0.05) (Figure 2A). As previously described, mir-660-5p had no effect on the migration of H1299 cells, due to the absence of p53 protein (Figure 2B). To further confirm the importance of mir-486-5p in the activity of mir-660-5p, proliferation after mir-660-5p over-expression and mir-486-5p inhibition was analyzed. As shown in Figure 2C, mir-660-5p significantly reduced A549 proliferation at 72 and 120 h post transfection compared to controls (40% of reduction in proliferation, *p* < 0.05), whereas the inhibition of mir-486-5p in the same cells resulted in a lower reduction of cell growth (21% of proliferation reduction, *p* < 0.05). As expected, no significant changes in H1299 proliferation were observed (Figure 2D). Successful inhibition of mir-486-5p levels after mir-660-5p over-expression is shown in Figure 2E.

### 2.3. MDM2 Silencing Induces Increased Expression Level and Activity of mir-486-5p in Lung Cancer Cells

In order to prove that mir-486-5p regulation is effectively due to mir-660-5p levels in cells and its activity on p53 pathway, MDM2, which is, as we recently demonstrated, a direct target of mir-660-5p in lung cancer cells, was silenced. A549 and H1299 cell lines were transiently transfected with a commercial MDM2 siRNA and then mir-486-5p modulation was assessed.

**Figure 3:**
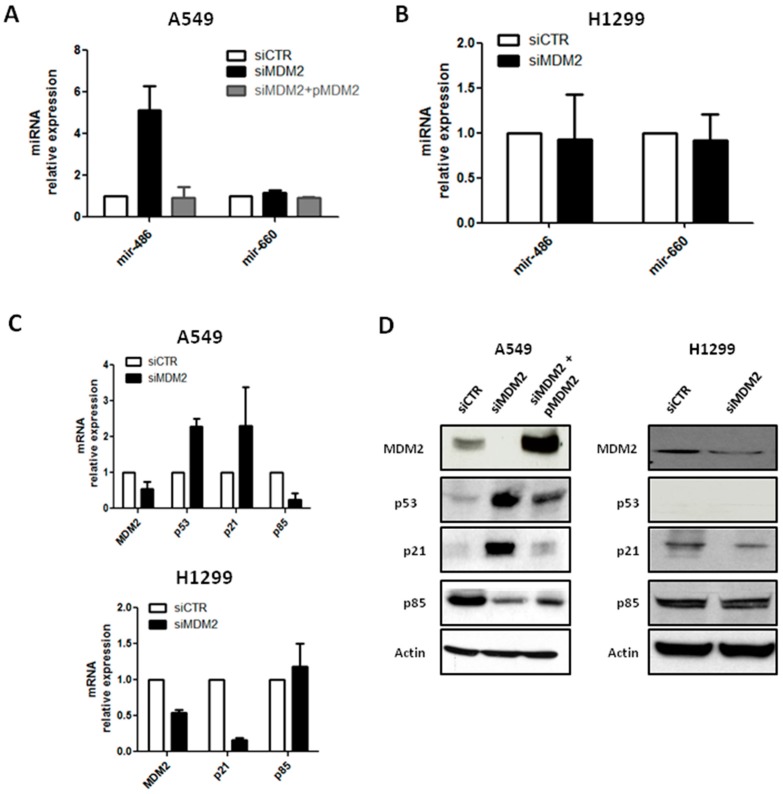
MDM2 down-modulation increased mir-486-5p levels and activity. (**A**,**B**) mir-486-5p and mir-660-5p expression after MDM2 silencing in A549 (**A**) and H1299 (**B**) lung cancer cells; (**C**) bar graphs showing transcript modulation in A549 (upper graph) and H1299 (lower graph) cells after down-modulation of MDM2; (**D**) representative Western blot bands of the MDM2/p53 axis and their downstream genes in A549 (left) and H1299 (right). All data are expressed as mean ± SEM (*n* = 2 for each cell).

Following siRNA-mediated MDM2 inhibition, an increase of mir-486-5p, of approximately six-fold compared to control, was observed in A549 cells (Figure 3A), whereas mir-486-5p up-regulation was not identified in H1299 (Figure 3B). mir-660-5p levels did not significantly change after MDM2 inhibition, neither in A549 nor in H1299 (Figure 3A,B). To confirm that siRNA transfection did not have any off-target effects, we added the MDM2 plasmid to MDM2-knockdown A549 cells. After MDM2 restoration we did not observe any significant mir-486-5p up-regulation (Figure 3A). Moreover, mir-660-5p levels were not affected by MDM2 replacement, as reported in Figure 3A.

Furthermore, in A549, after siRNA transfection, a significant reduction of MDM2 mRNA (75% compared to control; Figure 3C) was revealed and a decreased MDM2 protein expression was identified (Figure 3D).

Moreover, p53 levels, both as transcripts and proteins, were measured, and a significant increase of p53 expression was detected in MDM2 knockdown-A549 (2.3-fold increase for mRNA) (Figure 3C,D).

To demonstrate that p53 and mir-486-5p up-regulation after MDM2 silencing could regulate their downstream genes, mRNA and protein levels of p21^WAF1/CIP1^, a cyclin-dependent kinase inhibitor, were also analyzed in the same cells. A significant increase of p21^WAF1/CIP1^ levels were observed, either in transcript or in protein expression levels (Figure 3C,D). According to the mir-486-5p up-regulation, a down-modulation of p85 levels in real-time and Western blot analysis was detected (Figure 3C,D). The modulation of the MDM2/p53 pathways observed after MDM2 silencing was totally abrogated in A549-MDM2 knockdown cells after MDM2 restoration (Figure 3D).

Interestingly, down-modulation of MDM2 mRNA and protein was also visible in H1299, but without stimulation of p21 transcripts or proteins, and without any modulation of p85, indicating that functional p53 is critical for the regulatory effect of the mir-660-mir-486 network (Figure 3C,D).

Together, these data confirm that mir-660-5p is responsible for mir-486-5p positive modulation through its direct targeting of MDM2 in p53 wild-type lung cancer cells.

### 2.4. The mir-660-mir-486 Regulatory Pathway Is p53-Dependent

**Figure 4:**
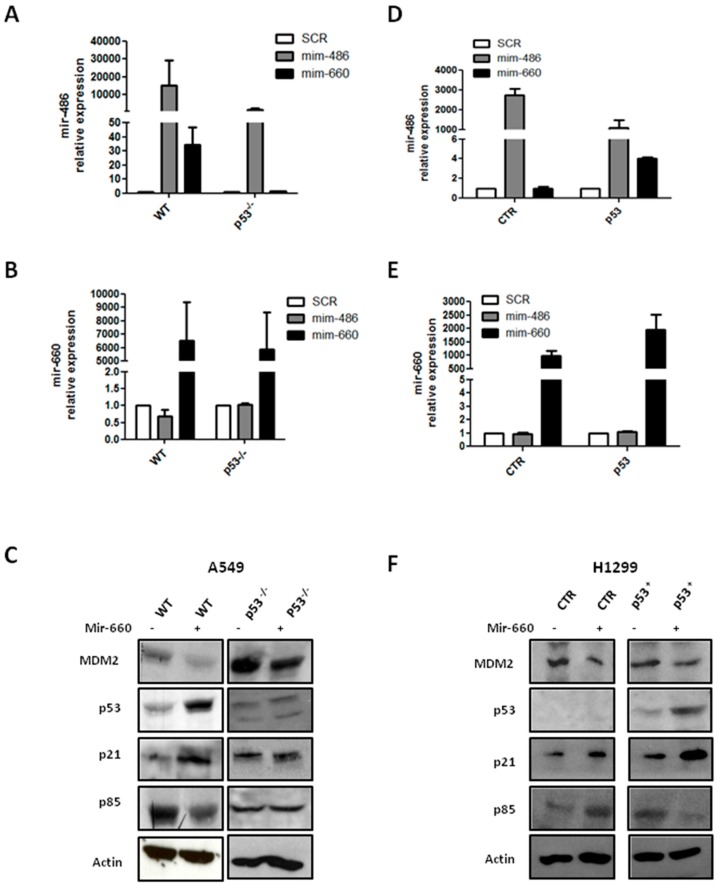
p53 is fundamental for mir-660-5p-dependent regulation of mir-486-5p. (**A**,**B**) Relative mir-486-5p (**A**) and mir-660-5p (**B**) expression in A549 WT and p53^−/−^; (**C**) MDM2/p53 axis and their downstream genes levels in A549; (**D**,**E**) relative mir-486-5p (**D**) and mir-660-5p (**E**) expression in H1299 CTR and p53^+^; (**F**) H1299-p53^+^ mir-660-5p over-expressing cells by Western blot analysis. All data are expressed as mean ± SEM (*n* = 2 for each cell).

Our data proved a positive modulation of mir-486-5p after mir-660-5p restoration in A549 (wild-type p53), but not in H1299 (homozygous partial deletion of the *TP53* gene resulting in a loss of expression of p53 protein), suggesting that a functional p53 protein is essential for mir-660-5p regulatory action on mir-486-5p. In order to verify this hypothesis, we evaluated mir-660-5p-mir-486-5p cross-talk in A549 knockdown for p53 (A549 p53^−/−^), obtained using Clustered Regularly Interspaced Short Palindromic Repeats CRISPR/cas9 technology, and in H1299 restored for p53 expression (H1299-p53).

mir-486-5p up-regulation, as illustrated by Figure 4A, after mir-660-5p over-expression was absent in A549 p53^−/−^ cells, compared to wild-type cells, whereas the expression level of mir-660-5p remained almost unaffected in both A549 wild-type and A549 p53^−/−^ cells (Figure 4B). Moreover, an effective MDM2 down-modulation with an increase of p53 expression in A549 wild-type cells treated with a mir-660-5p mimic was detected (Figure 4C). The functional role of mir-660-5p-mir-486-5p was confirmed by p21 up-regulation and p85 reduction in the same cells. Interestingly, p53 knockdown in A549 p53^−/−^ mir-660-5p over-expressing cells did not alter the expression of downstream genes, as shown by Western blot assay (Figure 4C).

On the other hand, restoration of p53 in H1299 cells led to an approximately four-fold increase of mir-486-5p, after mir-660-5p over-expression (Figure 4D). Again, the mir-660-5p expression level was similar in H1299-CTR and H1299-p53 (Figure 4E). Recovery of p53 protein in H1299-p53 cells was fundamental for the anti-tumoral activity of mir-660-5p; in particular, after mir-660-5p over-expression, in H1299-p53 cells an increase of p21 protein expression and p85 down-modulation was detected, as indicated by Western blot bands (Figure 4F).

These data prove that p53 is an essential component in the mir-660-5p and mir-486-5p interplay.

## 3. Discussion

It is becoming clear that small changes in miRNA expression may have large effects on cellular mechanisms, even if miRNAs only moderately suppress their target genes. Further evidence shows that miRNAs amplify their effects by regulating transcription of other miRNAs. An example of these mechanisms is the cross-regulation of miRNAs that are encoded within myosin heavy chain genes [32]. mir-709 was already described as a regulator of mir-15a/16 clusters at the post-transcriptional level in the nucleus of a mouse model [33].

Our results demonstrates that, in p53 wild-type lung cancer cells, mir-660-5p was able to indirectly regulate the expression of mir-486-5p, another miRNA that is de-regulated in aggressive forms of lung cancer, through the stabilization of p53. This finding provides further insight into mir-660-5p mechanism of action where a modest effect on MDM2 expression, already described by our group [29], dramatically reduces lung cancer growth in a p5-dependent manner.

Recent studies revealed interactions between p53 and miRNAs [21]. It was already demonstrated that p53 directly regulated the expression of tumor-suppressor miRNAs as the miR-34 family members [34], or mir-16 and mir-145, through a Drosha-mediated mechanism [35]. Interestingly, mir-29 was identified as another positive regulator of p53, through the repression of p85 [36]. In particular, in this work the reduction of p85 decreased PI3K/AKT activity resulting in reduced phosphorylation of AKT and MDM2, which, in turn, activated p53. This finding supports our results and indicates that mir-660-5p and mir-486-5p are critical players in the PI3K-–AKT-MDM2p53 network, a fundamental pathway in such cancers as leukemia, breast and lung tumors.

p53 is found inactivated in NSCLC by mutation or by MDM2 over-expression, which induces p53 elimination. Several works described MDM2 amplification in 7% human solid tumor, such as liposarcoma (50%–90%), osteosarcomas (16%), esophageal carcinomas (13%), and NSCLC (6%) [37].

In the past decade, reconstitution of p53-dependent pathways in tumor cells emerged as an effective therapeutic strategy [38] and, based on our previous published work, mir-660-5p replacement represents an alternative option to pharmacological molecules as nutlins [39] for lung cancer therapy. Furthermore, in this study, we showed that relatively small changes of mir-660-5p expression could potentially target several genes of redundant pathways, such as mir-486-PI3K/AKT signaling and, thus, potentially able to interfere with several pro-tumoral mechanisms.

In conclusion, we introduce an additional component to an already complex pathway: mir-486-5p is positively regulated by mir-660-5p expression, through the mir-660-5p-MDM2-p53 pathway, in lung cancer cell lines. In this context, mir-660-5p becomes even more interesting for its strong role as a tumor suppressor in lung cancer and, therefore, for its potential use in cancer therapy.

## 4. Materials and Methods

### 4.1. Cell Lines and Transfection

Human lung cancer cell lines, A549 and H1299, were obtained from the American Type Culture Collection (ATCC, LGC Standards). Cells were cultured in RPMI 1640 medium supplemented with 10% heat inactivated fetal bovine serum (FBS) and 1% penicillin-streptomycin (Sigma Aldrich, Saint Louis, MO, USA). Cells were transfected using mirVana miRNA mimics (Thermo Fisher Scientific, Waltham, MA, USA) or locked nucleic acid (LNA) inhibitor (Exiqon, Vedbæk, Denmark) (50 nM) according to the manufacturer’s instructions. pMDM2 was kindly provided by Scotlandi. p53 transfection was performed using p53 plasmid (Origene, Rockville, MD, USA) following the company’s protocol.

### 4.2. miRNA and mRNA Expression Analysis

Total RNA was extracted using a mirVana PARIS Kit (Thermo Fisher Scientific), following the manufacturer’s specifications, and then quantified with a NanoDrop 2000 system (Thermo Fisher Scientific). miRNA were retrotranscribed by using the TaqMan microRNA Reverse Transcription Kit and a TaqMan RT Primer Pool specific for miRNAs of interest, according to manufacturer’s instructions (Thermo Fisher Scientific). For gene expression analysis, reverse transcription was performed starting from 250 ng of total RNA. TaqMan microRNA assay (Thermo Fisher Scientific) and ready-to-use Assay on Demand (Thermo Fisher Scientific) were used with an Applied Biosystems 7900 System to quantify and analyze the level of mature miRNA and selected genes, respectively. MiRNA expression was normalized to the small nucleolar RNU48, and the *GAPDH* gene was used as the reference for sample normalization.

### 4.3. In Situ Hybrization (ISH) Analysis of miRNAs

miRNA in situ hybridization was performed on A549 and H1299 cell-blocks, as previously described by Gualeni et al. [40]. The protocol is based on a combination of double DIG-conjugated mirCURY locked nucleic acid (LNA) probes (Exiqon) and an automatic DAB-chromogenic detection system that, together, enable specific and sensitive detection of miRNAs.

Probes selected for ISH analysis are listed in Table 1. A scramble probe (sequence with no homology to any known miRNA or mRNA sequences) was used as the negative control.

Prior to use, each probe was pre-denatured at 90 °C for 4 min and diluted with ISH buffer (Exiqon) to final concentrations of 100 nM for miRNA specific probe and 40 nM for the scrambled-negative control probe.

In brief, Formalin-fixed, paraffin-embedded (FFPE) sections (5 µm thick) were first deparaffinized in xylene, rehydrated on alcohol at a descending scale, and treated with Proteinase-K (Sigma Aldrich) diluted 1:200, for 15 min, at 37 °C on a Dako Hybridizer (Dako, Glostrup, Denmark). Slides were washed in PBS 1X, dehydrated in alcohol at an increasing scale, and air-dried.

Samples were hybridized with the probe mixture for two hours in the Dako Hybridizer, at the specific probe hybridization temperature (Hybridization T = RNA T_m_ −30 °C) (Table 1). Slides were then stringently washed in 5× saline-sodium citrate (SSC), 1× SSC, and 0.2× SSC, at the hybridization temperature for 5 min each, and in 0.2× SSC at room temperature for 5 min. Following a wash in distilled water and one in Reaction Buffer 1× (Ventana Medical Systems, Oro Valley, AZ, USA), the miRNA expression was automated detected with the Ventana BenchMark ULTRA instrument using the OptiviewDAB Detection Kit (Ventana Medical Systems).

For image analysis, stained sections were examined by optical microscope and scanned with Aperio Scanscope XT (Leica Biosystems, Nussloch, Germany).

### 4.4. Functional Assays

Lung cancer cells were seeded in a 12-well plate at 2 × 10^5^ cells and proliferating cells were counted after 72 and 120 h by the trypan blue exclusion method (Sigma Aldrich).

For the migration assay, we used FluoroBlok Cell Culture Inserts (BD Biosciences, San Jose, CA, USA). Briefly, 10^5^ cells were seeded on the top chamber of the insert and RPMI plus 10% FBS was added to the bottom chamber and incubated at 37 °C and 5% CO_2_. After 24 h, migrated cells were fixed and stained with 4′,6-diamidino-2-phenylindole (DAPI). Migrated cells were counted using fluorescence microscopy. Migration graphs represented the number of migrated transfected cells versus control cells. Each experiment was performed in triplicate.

### 4.5. Western Blot Analysis

Proteins were lysed and extracted through incubation with radioimmunoprecipitation assay (RIPA) buffer (Sigma Aldrich). Bradford reagent was used to quantify the extracted proteins. About 40 μg of protein were separated by Nupage 4%–12% polyacrylamide gels (Thermo Fisher Scientific) and transferred to polyvinylidene difluoride (PVDF) membranes (GE Healthcare, Aurora, OH, USA). The membranes were blocked with 5% milk in phosphate-buffered saline with 0.05% Tween 20 (PBS-T) buffer and then incubated with the following primary antibodies: mouse anti-MDM2 (1:1000, Abcam, Cambridge, UK), p85 (1:1000 Cell Signaling), p53 (1:2500, Abcam, Cambridge, UK), p21 (Santa Cruz Biotechnology, TX, USA 1:1000), and rabbit anti-α-actin (1:2500, Sigma Aldrich), at 4 °C overnight. The membrane was washed with PBMS-T and then incubated for 1 h, at room temperature, with goat anti-rabbit or goat anti-mouse horseradish peroxidase (HRP)-conjugated secondary antibodies (1:2000, GE Healthcare). The membranes were developed using a chemiluminescence reaction (ECL, GE Healthcare). The data were analyzed using ImageJ software (NIH, Bethesda, MD, USA).

### 4.6. CRISPR/cas9 Protocol

A549 p53^−/−^ were obtained using a *TP53* human gene knockout kit according to manufacturer’s instructions (Origene, Rockville, MD, USA).

## Figures and Tables

**Table 1:** List of detection probes.

Probe	RNA T_m_ (°C)	T hyb (°C)	Probe Sequence
Scramble	87	57	GTGTAACACGTCTATACGCCCA
Hsa-mir-486-5p	92	62	CTCGGGGCAGCTCAGTACAGGA
Hsa-mir-660-5p	86	56	CAACTCCGATATGCAATGGGTA

T_m_: melting temperature; T hyb: hybridization temperature.
